# High dose piracetam on alcoholic cerebellar degeneration: A case report

**DOI:** 10.1192/j.eurpsy.2023.816

**Published:** 2023-07-19

**Authors:** G. Öksüz, M. Ergelen

**Affiliations:** Psychiatry, İstanbul Erenköy Mental Health and Neurological Diseases Training and Research Hospital, Istanbul, Türkiye

## Abstract

**Introduction:**

Chronic alcohol use is related to alcoholic cerebellar degeneration that is caused by B1 (thiamine) deficiency and associated with the degeneration of Purkinje cells in the cerebellar cortex.

**Objectives:**

This report aims to present a case with cerebellar ataxia and tremor due to alcoholic cerebellar degeneration that is unexpectedly regressed after starting piracetam infusion treatment in the inpatient Alcohol and Drug Addiction Research, Treatment and Education Center (AMATEM).

**Methods:**

We investigated the case prospectively. The patient was informed and consent was obtained.

A 57-year-old, divorced, retired, male with alcohol use disorder for 48 years (mostly high alcohol, cologne for 2 years) presented to our hospital with upper and lower limb tremors and balance problems for 2 years. Neurological examination revealed dysmetria, cerebellar tremor, hypoesthesia of lower extremities, ataxia so he was unable to tandem-walk. CIWA-Ar score:13. The blood test, including hemogram, biochemical, HbA1C, TFT, serum copper and ceruloplasmin levels, results were all normal. Diazepam 50 mg was started and titrated down by 5 mg per day and discontinued, 600 mg parenteral and 250 mg p.o thiamine initiated for 3 days and continued orally, propranolol 20 mg/day and ecopirin 100 mg/day were continued. Cranial CT, cranial MRI, and EMG were ordered.The patient was consulted to the neurologist for movement disorder.

**Results:**

MRI appearance of the cortical sulcus, fissure, cisterna, and cerebellar folia were obvious. The ventricular system was assessed as ecstatic secondary to atrophy. Therefore the alcoholic cerebellar degeneration was diagnosed and increased propranolol to 30 mg/day dose. Furthermore iv infussion piracetam was empirically started at secuencely dose of 60 g/day for 3 days, 45 g/day for 3 days, 30 g/day for 3 days. Upper and lower limb tremor disappeared and ataxa regressed after the treatment was arranged.

**Image:**

**Figure:**
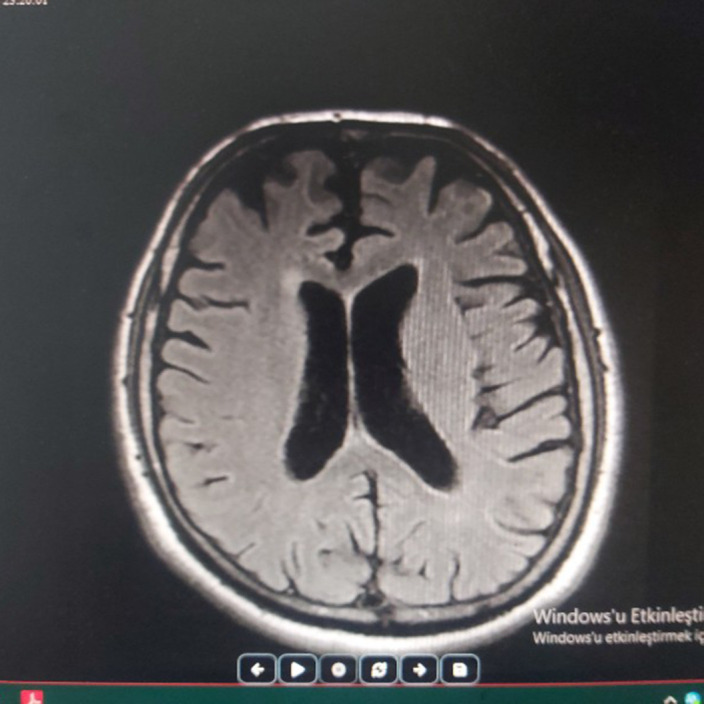

**Image 2:**

**Figure:**
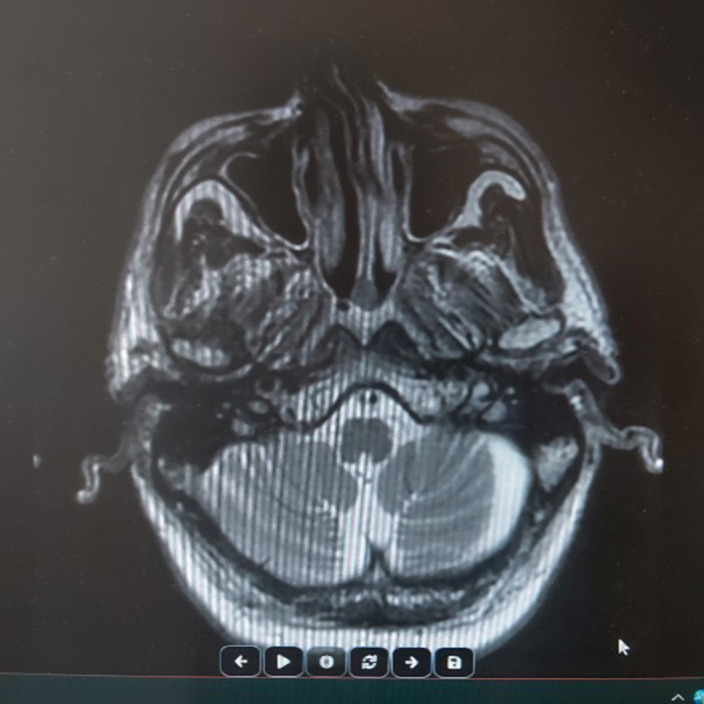

**Image 3:**

**Figure:**
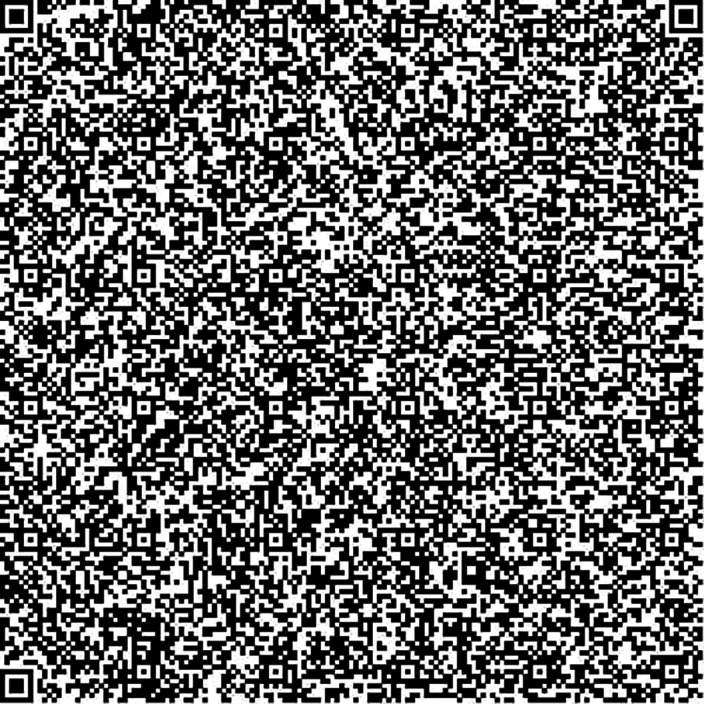

**Conclusions:**

Several authors proposed that serotonergic system among other neurotransmitters in the cerebellum might be affected in ACD. Recent studies investigating the use of buspirone in the treatment of ataxia has showed positive results (1-3).

The mechanism of action of the piracetam is unclear. Supposed that piracetam binds to the polar head groups of membrane bilayers and induces distinct changes in membrane structure. Its actions in the nervous tissue include indirect modulation of several neurotransmitter systems, neuroprotective and anticonvulsant effects and positive influence on neuronal plasticity.

In conclusion, we suggest that high dose piracetam has a potential antiataxic effect in ACD.

Cessation of drinking and nutritional supplementation are the only treatments available for ACD. However, gait does not improve in most patients. Pysical therapy, canes, walkers, and wheelchairs are helpful in maintaining mobility (4). Similarly, we observed that the patient’s need for care has been continuing.

**Disclosure of Interest:**

None Declared

